# Marine and Agro-Industrial By-Products Valorization Intended for Topical Formulations in Wound Healing Applications

**DOI:** 10.3390/ma15103507

**Published:** 2022-05-13

**Authors:** Ana-Maria Prelipcean, Andreea Iosageanu, Alexandra Gaspar-Pintiliescu, Lucia Moldovan, Oana Craciunescu, Ticuta Negreanu-Pirjol, Bogdan Negreanu-Pirjol, Raul-Augustin Mitran, Mariana Marin, Ugo D’Amora

**Affiliations:** 1 National Institute of R&D for Biological Sciences, 296 Splaiul Independentei, 060031 Bucharest, Romania; annastanciuc@gmail.com (A.-M.P.); andreea.iosageanu@gmail.com (A.I.); alex.gaspar@yahoo.com (A.G.-P.); moldovanlc@yahoo.com (L.M.); oana_craciunescu2009@yahoo.com (O.C.); 2 Faculty of Pharmacy, University “Ovidius” of Constanta, 1 Aleea Universității, 900470 Constanta, Romania; ticuta_np@yahoo.com (T.N.-P.); bnegreanupirjol@yahoo.com (B.N.-P.); 3 “Ilie Murgulescu” Institute of Physical Chemistry, Romanian Academy, 202 Splaiul Indepedentei, 060021 Bucharest, Romania; raul.mitran@gmail.com (R.-A.M.); mariana_marin12@yahoo.com (M.M.); 4 Institute of Polymers, Composites and Biomaterials, National Research Council, 80125 Naples, Italy

**Keywords:** *Rapana venosa* compounds, *Cladophora vagabunda*, grape pomace, marine and agro-industrial by-products valorization, wound healing, topical formulations

## Abstract

Over the past years, research attention has been focusing more on waste-derived, naturally derived, and renewable materials, in the view of a more sustainable economy. In this work, different topical formulations were obtained from the valorization of marine and agro-industrial by-products and the use of Carbopol 940 as gelling agent. In particular, the combination of extracts obtained from the marine snail, *Rapanosa venosa,* with *Cladophora vagabunda* and grape pomace extracts, was investigated for wound healing purposes. *Rapana venosa* has demonstrated wound healing properties and antioxidant activity. Similarly, grape pomace extracts have been shown to accelerate the healing process. However, their synergic use has not been explored yet. To this aim, four different formulations were produced. Three formulations differed for the presence of a different extract of *Rapana venosa*: marine collagen, marine gelatin, and collagen hydrolysate, while another formulation used mammalian gelatin as further control. Physico-chemical properties of the extracts as well as of the formulations were analyzed. Furthermore, thermal stability was evaluated by thermogravimetric analysis. Antioxidant capacity and biological behavior, in terms of cytocompatibility, wound healing, and antimicrobial potential, were assessed. The results highlighted for all the formulations (i) a good conservation and thermal stability in time, (ii) a neutralizing activity against free radicals, (iii) and high degree of cytocompatibility and tissue regeneration potential. In particular, collagen, gelatin, and collagen hydrolysate obtained from the *Rapana venosa* marine snail represent an important, valuable alternative to mammalian products.

## 1. Introduction

Skin tissue represents the largest human organ reaching ten percent of the total body mass. Its main role is acting as a protective barrier against the environment, but it is also responsible for sensory detection, fluid homeostasis, thermo- and immuno-regulation [1]. In physiological circumstances, after an injury, skin integrity should be easily restored by the human body with a minimal scar, through a complex and interactive process [2]. However, its self-regeneration ability can be seriously compromised under specific conditions, such as extensive skin loss, deep burns, chronic wounds, non-healing ulcers, and other diseases such as diabetes [2].

Considering that a series of factors may affect wound healing, a medical treatment is often necessary. In this scenario, over the past years, different wound care products have been developed to improve the quality of life. Since ancient times, owning to their therapeutic activities, availability and affordability with relative low cost, natural compounds have been commonly employed as emulsions and ointments in direct contact with skin [3]. Commonly, these treatments involved the uses of natural compounds obtained from different sources (i.e., herbal-derived and animal-derived compounds). Among the herbal-origin products, it is worth noting to cite herbs containing biologically active compounds (i.e., *aloe vera, Calendula officinalis*), while among the animal-origin compounds, honey and *propolis* certainly emerged with their known healing properties [4]. Nowadays, given the great attention on the environmental decline occurring worldwide, exploring the waste-derived, naturally derived, and renewable products, in view of a more sustainable and circular economy, has driven an outstanding research curiosity through the scientific community.

However, even though it is expected that natural compounds will assume a pivotal role in the healthcare, as they are a valuable origin of therapeutic substances for direct applications as topical wound healing agents, for the development of new classes of drugs with specific activities for each phase of the wound healing process they still highlight some limitations. If applied alone and not in combination with a natural or synthetic biomaterial wound dressing, they are not able to ensure an optimal treatment. Indeed, an ideal wound dressing should be able to provide a moist wound environment, offer protective role in secondary infections, remove wound exudate, and promote tissue regeneration [2]. Considering the above factors, modern wound dressings employ many biopolymers and bioactive compounds obtained from natural resources to develop different types of dressing biomaterials [5].

In particular, hydrophilic polymer networks, which confer an insoluble behavior to the polymeric system, are mainly employed in dry-to-moderately draining wounds to promote autolytic debridement in necrotic wounds and in granulating wounds [6]. Due to the composition and mechanical behavior, these biomaterials show properties which are similar to those of the natural extracellular matrix. In such a way, the smart dressing not only acts as the supporting material for cells in the tissue regeneration process but can also serve as the delivery platform of a bioactive compound [6]. Synthetic polymers (i.e., poly(acrylate acid) (PAA), poly(ethylene glycol) (PEG), poly(vinyl alcohol) (PVA), or carboxypolymethylene (Carbopol or Carbomer)) have been widely employed for wound healing application as they possess significant mechanical strength and durability [7,8,9]. However, natural polymers synthesized from plants, animals, and microbial sources (i.e., collagen, gelatin, chitosan, alginate, hyaluronic acid, or gellun-gum) and their modified derivatives are emerging as powerful platforms in wound healing treatment as they are non-toxic and biocompatible substrates equivalent to macromolecules recognized by the human body [10,11,12,13]. In this scenario, recent trends are moving towards the development of more specialized wound healing treatments involving the combined use of natural compounds, synthetic/natural biopolymers, and tissue engineering strategies [14].

Hence, the aim of the present study was to develop innovative topical formulations for efficient wound healing management. The main active components of the proposed formulations were derived from valorization of marine resources, *Rapana venosa* and *Cladophora vagabunda*, and the valorization of specific agro-industrial by-products, the grape pomace.

*Rapana venosa* is a predatory and invasive marine snail belonging to the *Muricidae* family which has a negative impact on different mussels and mollusks populations inhabiting the Black Sea [15]. Besides its nutritional value, when consumed as a seafood [16,17,18] *Rapana venosa* has been also reported to be a promising source of active biological compounds. Previous studies showed that amino acids, lipids, and proteins, extracted from the soft tissue of *Rapana venosa,* demonstrated wound healing properties and antioxidant activity [19,20,21]. Furthermore, a recent study from Gaspar-Pintiliescu et al. reported that the marine snail could represent an alternative source of collagen and its derivatives (e.g., gelatin and peptides) [22].

The interest in these naturally derived biopolymers in healthcare related sectors has had very fast growth in recent years. Collagen-based products obtained from marine organisms are considered compatible, pathogen free, and have no religious restrictions compared to mammalian collagen [23,24]. In addition, collagen extraction from by-products of fish processing industry, such as skin and scales, can significantly reduce environmental pollution. Besides different species of fish, other marine sources have also been reported to contain collagen and gelatin, such as sponges [25,26], jellyfishes [27,28], squids [29], and snails [22,30]. In particular, marine collagen and its derivatives have been reported to promote wound healing in vitro by improving cell proliferation and migration, up-regulating the expression of chemotactic factors (i.e., β-Fibroblast growth factors (FGFs) and Transforming growth factor (TGF)-β1), or important growth factors (i.e., Vascular endothelial growth factor (VEGF)) and stimulating the recruitment of inflammatory cells. Different formulations of collagen-based products, applied on wounded animal models, showed an increased re-epithelization rate, wound closure, and granular tissue deposition compared to control groups [24,31].

*Cladophora vagabunda* is a green alga found in abundance on the coastal waters of the Romanian Black Sea shore. Previous studies showed that *Cladophora vagabunda* is an important source of volatiles compounds, fatty acids, terpenoids, sterols, phenols, carbohydrates, vitamins, and minerals, with potential for developing new therapeutics or production of biodiesel [32,33,34].

Grape pomace, which is the solid waste generated from pressing and fermentation processes in the vinification of grape varieties, is an important source of fibers, polyphenolic compounds, soluble sugars, and minerals with strong antioxidant and antimicrobial effects [35]. Topical application of grape pomace powder from Cabernet Sauvignon variant on excision wounds created on rats has been reported to accelerate the healing process [36].

However, the combination of algal extracts with marine collagen or gelatin from *Rapana venosa* and grape pomace extract has not been investigated yet for wound healing purposes.

## 2. Materials and Methods

### 2.1. Materials and Chemicals

*Rapana venosa* specimens and *Cladophora vagabunda* algae were collected during the summer period from the Romanian seacoast of the Black Sea between the 2 Mai and Vama Veche areas. The snails were washed with cold distilled water (*di*H_2_O) and stored at −20 °C until use. Similarly, the marine algae were washed with cold *di*H_2_O and dried at room temperature (T_room_).

Red grapes pomace (*Vitis vinifera L*.) Mamaia variety was provided by Murfatlar Research Station for Viticulture and Oenology, Constanta County, Romania.

Mammalian gelatin from pig skin (Type A) was purchased from Sigma-Aldrich (Saint Louis, MO, USA), as well as all the chemicals, unless otherwise specified.

### 2.2. Preparation and Characterization of Naturally Derived Extracts

**Preparation of *Rapana venosa* extracts**. The soft tissue of marine snails was removed from the hard shell, washed with *di*H_2_O, and cut into small pieces (2–5 mm). The cleaned tissue was subjected to alkaline pre-treatment with 0.5 M sodium hydroxide (NaOH) solution in a ratio of 1:10 (*w/v*) at T_room_ for 24 h in order to remove non-collagenous proteins.

*Marine Gelatin extraction.* Gelatin was extracted using acidic treatment, as previously described [22]. Briefly, the pre-treated tissue was extracted with 0.5 M acetic acid solution (1:10, *w/v*) at T_room_ for 24 h. After centrifugation at 8000× *g* for 40 min, the sample was heated at 60 °C. The resulting gelatin solution was dialyzed *di*H_2_O, and freeze-dried at −40 °C for 48 h.

*Marine Collagen extraction.* Collagen extraction was performed according to an adapted protocol from Gaspar-Pintiliescu et al. [37]. The pre-treated tissue was extracted with 0.5 M acetic acid solution (1:15, *w/v*) at 4 °C for 48 h. After centrifugation at 5000× *g* for 20 min, the supernatant was precipitated with 0.7 M sodium chloride (NaCl). The precipitate was collected after centrifugation at 5000× *g*, for 20 min, solubilized in 0.1 M acetic acid, and dialyzed against *di*H_2_O. All processes were carried out at 4 °C. The obtained collagen solution was freeze-dried at −40 °C for 48 h.

*Collagen Hydrolysate.* Collagen hydrolysate was obtained by enzymatic treatment. Briefly, samples of *Rapana venosa* collagen were dissolved in 0.2 M phosphate buffer, pH 6, containing papain with an enzyme substrate ratio of 25:1 (*w/w*), at 55 °C, for 6 h. The enzyme was inactivated by heating at 100 °C for 10 min. The resulting hydrolysate was then filtered, concentrated by centrifugal ultrafiltration at 7500× *g* for 25 min using filter units with cellulose membranes of 3000 Da M_W_, cut off, and dried at −40 °C.

**Preparation of *Cladophora vagabunda* ethanolic extract**. Grinded algae (10 g) were mixed with 100 mL ethanol/water solution (70/30, *v/v*) and placed in an ultrasonic bath for 30 min at 30 °C. Then, the extract was centrifuged at 3000× *g* for 15 min, and the resulting solution was filtered through Whatman filter paper to remove any remaining residues. The algal extract was kept at −4 °C until use.

**Preparation of grape pomace ethanolic extract**. Dried pomace (10 g) was placed into a volumetric flask (100 mL) and 70% aqueous ethanolic solution was added up to the mark. The extraction was carried out for 12 days, at T_room_, in the dark, with periodic stirring. The pomace extract was then filtered and stored at −4 °C until use.

**Characterization of marine-derived extracts.** Ash and moisture content of marine-derived extracts were analyzed at 600 °C and 120 °C, respectively, according to AOAC standard methodology [38]. Dry substance (DS, %) content was calculated by subtracting the moisture percentages from the weight of the original sample. Total protein content was determined using the Lowry protein assay and bovine serum albumin (BSA) as standard in the range of concentration of 0–10 mg/mL.

### 2.3. Preparation of Topical Formulations

**Figure 1:**
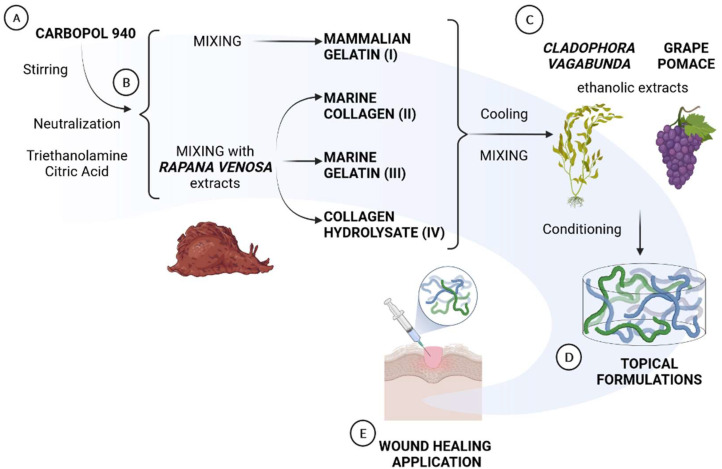
Schematic representation of the sequential steps in the preparation of the four different formulations: (**A**) Carbopol 940 dispersion in water, neutralization and stabilization; (**B**) mixing with mammalian gelatin (Formulation 1) or with *Rapana venosa* extracts (Formulations II, III and IV); (**C**) combination of the three formulations with *Cladophora vagabunda* and grape pomace extracts; (**D**) cooling and realization of the final formulations; (**E**) characterization of the formulations.

The sequential steps of preparation are indicated in Figure 1A–E. For the preparation of the different formulations, a specific amount of an acrylic acid polymer, Carbopol 940 (0.5 g), was dispersed in purified water (100 mL) at 40 °C and was mixed by a mixer at 1200 g for 30 min (Figure 1A). Carbopol 940 was selected as the thickener agent due to its easy and fast dispersion properties at T_room_, its wide use in the pharmaceutical, cosmetic, and dermocosmetic areas for the development of topical formulations containing hydroalcoholic extracts [39,40], and because it provides gels with good rheological properties. Furthermore, it is biodegradable, bioadhesive, biocompatible, and it has non-irritant properties [40]. The neutral pH was obtained by adding a required amount of triethanolamine (0.75 g), an organic amine, to gain a transparent gel and to obtain an adequate semisolid formulation for skin administration. Furthermore, in order to maintain the stability of active ingredients, a preservative, citric acid (0.75 g), was added to all formulations. The formulations were prepared according to the quali-quantitative formula described in Table 1. After neutralization, the three extracts (marine collagen, marine gelatin, and collagen hydrolysate) from *Rapana venosa* were added, obtaining formulation II, III, and IV, respectively. Formulation I employed mammalian gelatin as further control (Figure 1B). After cooling down the temperature, mammalian gelatin and each protein from *Rapana venosa* were combined with *Cladophora vagabunda* and grape pomace extracts in a ratio of 1:2:2 (*w/w/w*) under stirring (Figure 1C). Final formulations were prepared in triplicate for further analyses (Figure 1D,E).

Table 1 summarizes the different formulations with related composition and nomenclature.

### 2.4. Physico-Chemical Characterization of the Topical Formulations

**Organoleptic evaluation**. The formulations were analyzed for smell, homogeneity, color, and consistency by visual inspection.

**Thermal stability evaluation**. The thermal stability of the different formulations was assessed by thermogravimetric analysis (TGA). TGA coupled with differential thermal analyses (DTA) was carried out using a Mettler Toledo TGA/SDTA851e thermogravimeter, under 80 mL min^−1^ synthetic air atmosphere, at a heating rate of 10 °C min^−1^.

**Antioxidant capacity.** In order to evaluate the antioxidant potential of the different formulations, cyclic voltammetry (CV) and Differential Pulse Voltammetry (DPV) were used. The voltammetric measurements were performed in a three electrochemical cell using a potentiostat-galvanostat Autolab 302N controlled by GPES software. In particular, the electrochemical cell consisted of a glassy carbon electrode (GCE, 3 mm diameter, an auxiliary electrode consisting of a glassy carbon rod and Ag/AgCl (Metrohm) as a reference electrode. Before measurements, the surface of GCE was polished with 1–0.05 µm alumina powder, then washed with doubly *di*H_2_0 and stabilized by sweeping the potential between 0.1 V and 0.95 V for 5 consecutive scans with 50 mV/s in 0.1 M phosphate buffer pH 7. The stock solutions were obtained by dissolving of 0.1 g of each sample in 5 mL ethanol in an ultrasonic bath 20 min at T_room_. In order to assess the 2,2-Diphenyl-1-picrylhydrazyl (DPPH) radical scavenging capability, 2 mL of sample stock solutions were added to 8 mL of a solution containing 0.02 mg/mL DPPH radical in ethanol–phosphate buffer solution pH 7 (1:1 *v/v*). The sample was subjected to electrochemical measurements after dark incubation for 60 min. Similar procedure was performed in order to determine the redox properties by electrochemical methods, when 2 mL of stock solutions were diluted with 8 mL of 0.1 M phosphate buffer pH 7.0. The antioxidant activity (AA%) was calculated relative to DPPH from the anodic peak currents, according to Equation (1):


AA (%) = (I_DPPH_ • I_DPPH-SAMPLE_)/I_DPPH_• × 100 (1)

where: I_DPPH_• and I_DPPH-SAMPLE_ are the anodic peak currents of DPPH• and of DPPH•, respectively, in the presence of sample.

### 2.5. Biological Characterization of the Formulations

**In vitro cell culture.** Biological assays were performed using a stabilized cell line of rat fibroblasts NCTC clone L929 (ATCC) for cytocompatibility test, and human keratinocytes (HaCaT, ECACC via Addex Bio) for wound healing test. NCTC clone L929 derived from mouse connective tissue (normal subcutaneous areolar and adipose tissue of 100 day old male C3H/An, passage number: 565) with a morphology of fibroblast, while HaCaT derived from human keratinocytes (in vitro spontaneously transformed keratinocytes from histologically normal skin of male Caucasian of 62 years old). NCTC clone L929 cells were cultivated in Minimum Essential Medium Eagle (MEM) growth medium, supplemented with 10% fetal bovine serum (FBS) and 1% antibiotic solution (streptomycin 100 mg/mL and penicillin 100 U/mL), and maintained at 37 °C in a humid atmosphere with 5% CO_2_ and 95% air humidity. HaCaT cells were cultivated in RPMI growth medium (Biochrom, Berlin, Germany), supplemented with 10% FBS and 1% antibiotic solution (streptomycin 100 mg/mL and penicillin 100 U/mL), and maintained at 37 °C in standard conditions.

**Indirect cytotoxicity assay.** The method of the indirect contact was used according to ISO 10993:5 (Biological evaluation of medical devices). For each sample, a stock solution of 10 mg/mL was prepared in MEM supplemented with 10% FBS at 37 °C for 24 h. Prior to the in vitro evaluation, the extracts were diluted in order to obtain different concentrations ranging from 10–500 µg/mL. The solutions were sterile filtered with 0.22 µm Millipore membranes.

Fibroblast cells were seeded in 96 well plates at a cellular density of 4 × 10^4^ cells/mL and after 24 h, time necessary for cell adhesion, the growth medium was removed and different concentrations of conditioned media from the four formulations (10, 50, 100, and 500 µg/mL) were added. Cells were maintained in standard conditions for 24 and 48 h, and afterwards the MTT (Sigma, Steinheim, Germany) test was performed according to the manufacturer’s protocol. Briefly, cells were incubated with a 0.25 mg/mL MTT solution for 3 h at 37 °C in a humid atmosphere. The formazan crystals formed in the active metabolic cells were dissolved in acetic acid and the absorbance read at 570 nm with a Mithras LB 940 microplate reader (Berthold Technologies, Bad Wildbad, Germany). The results were reported as viability percentages relative to the control (untreated cells) considered to have a 100% viability.

**Wound healing evaluation, Migration potential assay.** In order to evaluate the influence of the different formulations on the migration and repair ability of an injured cellular monolayer, the Scratch assay was used. Briefly, HaCaT were seeded in 24 well plates at a cellular density of 1.5 × 10^5^ cells/well and cultivated in RPMI growth medium at 37 °C. After 24 h, a perpendicular linear wound (scratch) was created by means of a 200 µL sterile pipette tip on the cellular monolayer. A blank line perpendicular to the scratch, painted under the bottom of the plate, ensured the recording of the same wound area per well. After a gentle PBS wash for cellular debris removal, the formulation at the concentration of 100 µg/mL, which was previously sterile filtered, was added, and cells were maintained in standard conditions at 37 °C for 24 h.

After the scratch, wound closure was monitored collecting digitalized images at beginning of the experiment (time 0—immediately after the scratch) and after 24 h in order to evaluate the migration status of the cells and the coverage of the wounded area. To estimate cell migration, images of two sites per well through an inverted microscope (Axio Star Plus) and digital camera (Carl Zeiss, Jena, Germany) (10× objective) were captured. The wound areas were analyzed using ImageJ 1.51 software for quantitative analysis of the wound closure percentage, according to Equation (2). Four replicates per condition were used and three experiments were done.


Repair rate of scratch (%) = (A_To_ − A_Th_)/A_T0_ × 100 (2)

where A_T0_ corresponds to the whole wound taken immediately after the scratch, and A_Th_ corresponds to the whole wound taken after 24 h.

**Antimicrobial potential assessment.** The antimicrobial activity was assessed via a modified version of the diffusimetric antibiogram method Kirby–Bauer on pathogenic strains, specific for skin and mucous membranes, Gram-positive bacteria (*Staphylococcus aureus*, ATCC 25923), Gram-negative bacteria (*Escherichia coli*, ATCC 25922), and fungal (*Candida albicans*, ATCC 10231), respectively. Briefly, standardized microbial suspensions corresponding to 0.5 McFarland density were prepared from fresh (24 h) solid cultures of *Staphylococcus aureus* grown on trypticase soy agar (TSA) nutrient medium by aerobic incubation at 37 °C. The bacterial inoculum was added to give a final concentration of 1 × 10^8^ colony forming units per mL (CFU/mL) and 10 μL of each formulation at the concentration of 100 µg/mL was incubated for 24 h on inoculated agar plates. The diameter of the growth inhibition zones was monitored for three independent measurements.

### 2.6. Statistics and Data Analysis

For each experiment, results are presented as mean ± standard deviation (SD). For cytocompatibility and wound healing analyses, statistical analysis of variance of the means compared to untreated samples was assessed by two-way and one-way ANOVA, respectively, followed by Tukey’s post-hoc test, by using GraphPad Prism software (version 7.0). Different levels of significance were considered 95–99.9999% (* *p* < 0.05, # *p* < 0.001; ° *p* < 0.0001) among the different results.

## 3. Results and Discussions

### 3.1. Characterization of Naturally Derived Extracts

In the present study, collagen from the soft tissue of *Rapana venosa* was obtained according to the following steps: removal of non-collagenous proteins, extraction by chemical method with acetic acid and salt precipitation with NaCl, while gelatin was obtained by heat extraction with acetic acid. Both extracts were previously characterized, presenting a good cytocompatibility, promoting cell-induced capacity and lacking irritant potential when tested in vitro and compared with mammalian collagen and gelatin [22]. Collagen hydrolysate was obtained by papain hydrolysis of collagen samples. Papain is a plant-derived protease that was also successfully used in preparation of collagen hydrolysates derived from yellowfin tuna (*Thunnus albacares*) skin and Malaysian jellyfish, *Rhopilema hispidum* [41,42]. *Cladophora vagabunda* and pomace extracts were obtained using 70% ethanolic solution. This extraction method is preferred due to non-toxic nature of the solvents and is frequently used for recovering higher concentrations of analytes, especially polyphenols [43]. Previous studies reported that *Cladophora vagabunda* extracts containing polyphenols presented antioxidant and antimicrobial properties against two bacterial strains *Staphylococcus aureus* and *Escherichia coli* [44], while in the case of grape pomace from Mamaia variety it was shown that antioxidant activity of the ethanolic extract positively correlated with its total phenolic content [45].

The analysis of biomasses was carried out to determine dry substance (DS), ash-mineral substance, and total protein content in both marine sources: *Rapana venosa* and *Clodophora vagabunda*. The main biochemical properties are reported in Table 2. For *Rapana venosa*, the ash content and DS were low as the Asian marine gasteropods were collected during summer. Indeed, it was clearly reported that dry weight and ash content drop their lowest level during the spawning period, which falls between May and August. This result was confirmed by other previous studies [46,47]. The analysis of the biochemical composition indicated a high concentration of protein in *Rapana venosa*. Indeed, the high nutritional value of the marine organism is given by the high concentration of protein in the marine snail, as confirmed by the specialized literature [47]. In the present study, the total protein content of *Rapana venosa* was 56.87% comparable to the values found in the literature [22].

Total protein content of *Clodophora vagabuda* was 38.79%, higher than that found for other *Clodophora* microalgae, which showed a value ranging from 26.8% and 27.8% [48] or *Clodophora glomerata*, which was 14.45% [49]. Indeed, *Rapana venosa* and *Cladophora macroalgae* contain a high number of proteins and feature high moisture (around 60–90%) [50].

### 3.2. Physico-Chemical Characterization of the Formulations

Considering the biocompatibility of *Rapana venosa* extracts and their ability to promote cell adhesion [22] alongside with the antioxidant and antimicrobial potential of *Cladophora vagabunda* [33] and pomace extracts [35,44], these molecules were embedded in a formulation intended for wound healing application. Different formulations were prepared using Carbopol 940 as gelling polymer.

The macroscopic evaluation of the formulations showed satisfactory organoleptic characteristics in terms of odor, color, and appearance, indicating a smooth, homogenous, and slightly turbid, colored texture, associated with the algal and pomace extracts. These properties are very important to obtain good patient acceptance.

**Figure 2:**
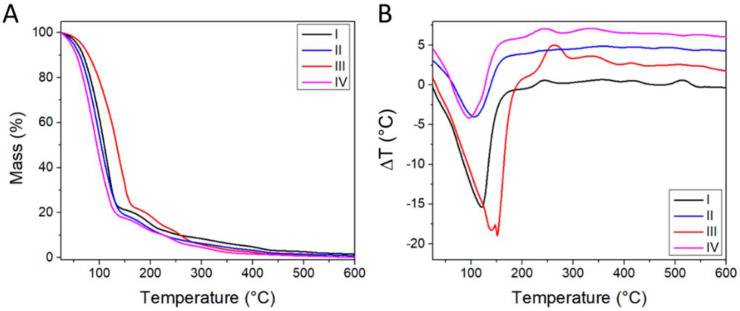
(**A**) Thermogravimetric Analysis (TGA) and (**B**) Differential Thermal Analyses (DTA) results for the different formulations. Each curve in (**B**), with the exception of formulation I, is shifted by +2 °C with respect to the previous one, for clarity.

From the thermogravimetric analyses, the formulations showed a loss of mass in the temperature range between 25 and 150 °C caused by water and volatile components’ (i.e., solvents) evaporation, associated with an endothermic effect. The combustion of organic components took place in the temperature range between 150 and 550 °C in at least three distinct steps, associated with exothermic effects (Figure 2). The thermal behavior of the four formulations was similar to that of Carbopol 940, with the exception of the solvent evaporation step (Supplementary Information, Figure S1). Representative samples of formulations I and III were dried at 40 °C for 14 days and remeasured by TGA (Supplementary Information, Figure S1). The water content decreased to 10–15% w, while the same thermal behavior was observed above 100% w. The thermal degradation of the dried formulations starts at ~140 °C, which is lower than the 180 °C temperature in the case of pure Carbopol 940 (Supplementary Information, Figure S1C). However, since the formulations are envisioned for body temperature applications, this decrease in thermal stability is not expected to significantly affect their potential applications.

The organic fraction and inorganic residue at 600 °C were computed based on the TGA data (Table 3).

**Figure 3:**
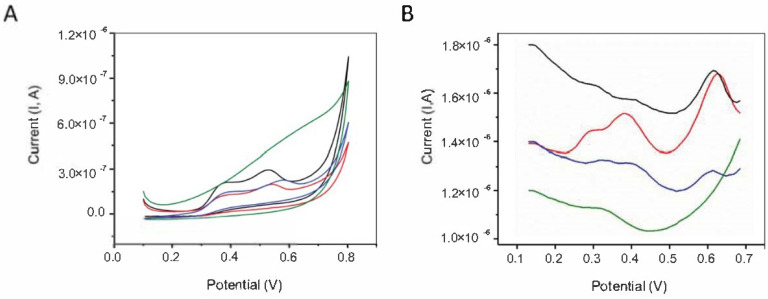
(**A**) Cyclic Voltammetry (CV) and (**B**) Differential Pulse Voltammetry (DP) of formulations: I (black line), II (red line), III (blue line), and IV (green line) in ethanol (0.1 M phosphate buffer of pH 7 (1/1 *v/v*)) at glassy carbon electrode (GCE). Scan rate 50 mV/s.

The antioxidant properties of different formulations have been evaluated by electrochemical analyses (CV and DPV) and by determination of DPPH radical scavenging capacity. The electrochemical methods are attractive tools for the determination of antioxidant activity owing to their simplicity, sensitivity, selectivity, low probe consumption, and rapidity of analysis. Moreover, the electrochemical signal is not affected by the sample complexity and by the turbidity. The representative cyclic voltammograms exhibited two anodic peaks situated at 0.37 V, 0.52 V, and 0.54 V, for the formulations I and II, whilst for the formulation III they were slightly displaced toward more electropositive potentials, i.e., 0.39 V and 0.58 V, respectively (Figure 3). In the case of formulation IV, only a broad peak at about 0.55 V was observed. The shifting of peak potentials could be probably due to the nature of polymer matrix used for the immobilization of bioactive compounds. The cyclic voltammogram of Carbopol 940 in ethanolic phosphate buffer of pH 7 was also performed (Supplementary information, Figure S2). The anodic current starts to increase after a potential of 0.3 V but a well-defined anodic peak cannot be observed for Carbopol 940. The anodic peaks of formulations I-IV indicated the presence of antioxidants belonging from different classes such as polyphenols and carotenoids. The low oxidation potentials revealed the capability of the bioactive compounds of formulations (I–IV) to donate electrons and to exhibit antioxidant activity. On the other hand, the absence of any peak on reverse scan indicated that the oxidized compounds were not involved in subsequent electrochemical reduction reaction [51]. Despite information regarding the reversibility of redox processes, CV has limitations with respect to peak resolution. Accordingly, DP voltammograms of the formulations (I–IV) were recorded within the range between 0.1 V and 0.7 V with a step potential of 9 mV and a modulation of amplitude of 0.03 V. The three anodic peaks located at approximately 0.28 V, 0.38 V, and 0.62 V underlined the antioxidant properties in aqueous buffered solutions and demonstrated that DPV is more sensitive than CV. The presence of an only weak anodic peak on the DP voltammogram of formulation IV suggested a lower concentration of antioxidant species, probably due to the consumption by O_2_ dissolved in aqueous buffered solutions.

However, all the formulations exhibited antioxidant activity due to their content of antioxidants, which can scavenge the DPPH radicals. In particular, the antioxidant potential is mediated by the ability of the bioactive compounds contained in the vegetal extracts (algae and pomace) to donate hydrogen atoms to DPPH. The voltammetric measurements indicated that the DPPH scavenging capacity varied from 20% to 52.49%, with the highest value obtained for formulation III (Table 3). The unexpected superior DPPH scavenging capacity observed for the formulation III, characterized by the highest redox potentials, could be assigned either to the synergistic effects of polymer matrix or to the different radicals quenching rates.

### 3.3. Biological Characterization of the Formulations

**Figure 4:**
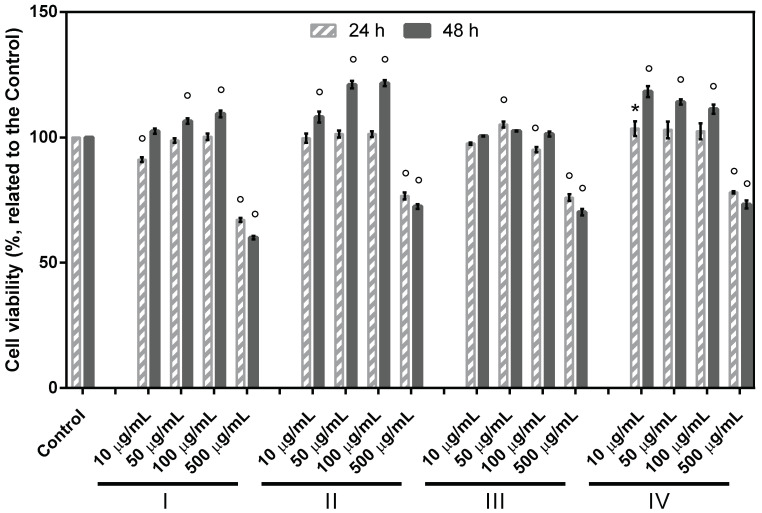
Viability of fibroblasts (NCTC clone L929, ATCC) cultivated in the presence of different concentrations of formulations I, II, III, IV, evaluated by MTT test at 24 and 48 h. Data represent the mean ± SD (*n* = 3). (* *p* < 0.05; ° *p* < 0.0001 compared to untreated control).

**Cytocompatibility analysis.** Non-cytotoxicity, biocompatibility, mild processing conditions, and physico-chemical properties similar to the natural tissues are among the major features which make collagen and gelatin suitable for skin tissue regeneration. In view of this, the basic cytocompatibility of the different formulations was assessed by evaluating the metabolic activity of rat fibroblasts NCTC clone L929. Results revealed that all the four tested formulations were biocompatible at both time points (Figure 4). Cell viability was >90% for the concentrations ranging between 10–100 µg/mL. Furthermore, the formulations II and IV containing gelatin and collagen hydrolysate from *Rapana venosa*, respectively, supported cell proliferation; higher cell viability percentages were registered for the first three tested concentrations (109.41–102.54%), compared to untreated control (100%), after 48 h.

**Figure 5:**
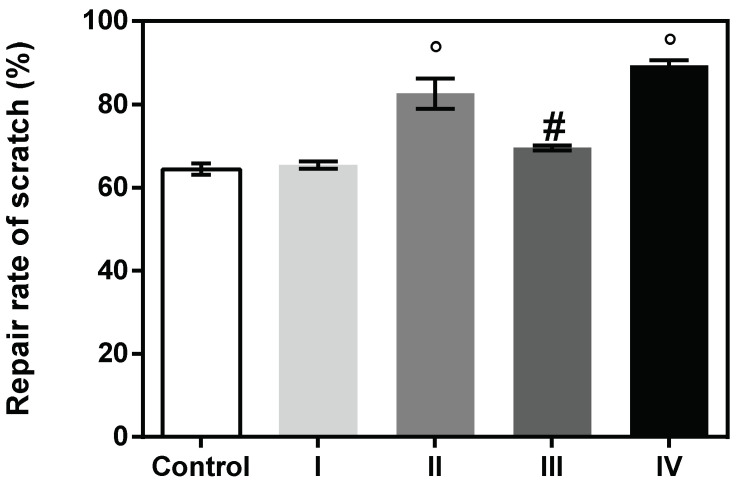
Repair rate of scratch (%) by cellular migration of human keratinocytes (HaCaT, ECACC) assessed by means of ImageJ 1.51 software. Data represent the mean ± SD. (# *p* < 0.001; ° *p* < 0.0001) compared to control.

**Figure 6:**
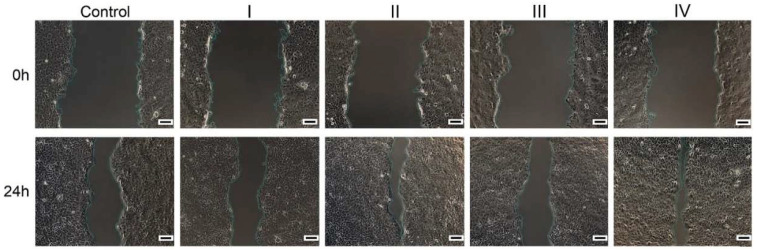
Optical microscopy images of wounded human keratinocyte monolayers (HaCaT, ECACC) (Control) and treated with the four formulations at time 0, immediately after the scratch, and after 24 h of cultivation. Scale Bar = 100 µm.

**Wound healing potential**. Based on the cytocompatibility data, the 100 µg/mL concentration was selected for a wound healing test. Results indicated that the repair rate of scratch (%) was higher for the treated cells when compared to the control, suggesting a beneficial effect of the formulations on the healing process. Hence, the highest closure area was observed for cells treated with formulation IV (89.35%), containing collagen hydrolysate, which stimulated a high rate of cell migration, followed by formulation II (83.75%) containing *Rapana venosa* gelatin. Mammalian gelatin and marine collagen-based formulations I and III presented a lower effect on cell migration (Figure 5 and Figure 6).

Keratinocyte’s migration was observed for all the conditions tested, and formulations II, III, and IV proved to be efficient in stimulating the closure rate of the wounded cell monolayer. The most powerful formulation seems to be formulation IV with a considerable percentage of migrated cells. The positive effect on tissue regeneration can be explained by a synergic activity of the different components embedded in the formulation. Moreover, the collagen hydrolysate from *Rapana venosa* presented the highest regenerative potential, in accordance with previous studies indicating that collagen hydrolysate from *Oreochromis nilocitus* was a suitable microenvironment to diminish the inflammatory phase duration, promoting wound healing [52,53]. Furthermore, in vivo tests on protein extracts from *Rapana venosa* showed that the samples reduced inflammatory infiltration, stimulated dermal and epidermal regeneration, and promoted angiogenesis, overall accelerating the time of healing from 22–24 days to 12–18 days [20]. The results suggested that marine gelatin and hydrolysate containing bioactive compounds of lower molecular mass could be more effective signaling molecules than large polymers such as marine collagen or mammalian gelatin. In addition, previous studies have shown that *Cladophora vagabunda* extracts containing bioactive compounds, such as tocopherol, carotenoids, and polyphenols, exhibited antioxidant and anti-inflammatory activity, which could accelerate the wound healing process [54]. Regarding formulations II and III, a good in vitro cytocompatibility with HaCaT cells and an increased proliferation and cell adhesion capacity for gelatin obtained *Rapana venosa* have been also confirmed [22].

**Antimicrobial activity.** The interference with the microbial growth was monitored and the results are summarized in Table 4. The experimental data showed that all four formulations exhibited a clear antibacterial activity against both Gram-negative *Escherichia coli* and Gram-positive *Staphylococcus aureus*. However, the antifungal properties were more limited and moderate for all tested formulations against the growth and multiplication of *Candida albicans*. *Escherichia coli*, the Gram-negative bacterial strain, proved to be more sensitive to the bioactive principles incorporated in the formulations, with larger growth inhibition zones when compared to the ones developed on Gram-positive *Staphylococcus aureus*. This effect can be correlated with the structural features of the bacterial species used in the study, respectively, the simpler cell wall architecture specific to Gram-negative strains that could be associated with an increased permissiveness to bioactive natural compounds.

The clear antibacterial activity of all the formulations could be due to the effect of bioactive compounds present in grape pomace and algal extracts, but also to their incorporation in Carbopol 940, which facilitated their gradual diffusion in the growth medium. Previous studies reported that *Cladophora vagabunda* extracts containing polyphenols presented antimicrobial properties against both bacterial strains of *Escherichia coli* and *Staphylococcus aureus* [44]. Grape pomace extract presented significant influence on the bacterial growth of *Staphylococcus aureus* and *Escherichia coli* [55].

## 4. Conclusions

In this study, different formulations were obtained from sustainable bioresources and industrial wastes with low environmental impact by marine sources valorization, collagen, gelatin, and collagen hydrolysate from *Rapana venosa* and ethanolic extracts from *Cladophora vagabunda*, respectively. Mamaia variety grape pomace, specific to the Black Sea region, was successfully added to the compositions. Despite the limitations of the current research, the following conclusions were reached:

- all the tested formulations exhibited a good conservation and thermal stability over time,
- they showed a neutralizing activity against free radicals,
- they were characterized by a high degree of cytocompatibility and tissue regeneration potential.

The present results suggest that collagen, gelatin, and collagen hydrolysate obtained from the *Rapana venosa* marine snail represent an important, valuable alternative to mammalian products. Furthermore, the different formulations could be employed as valuable candidates for smart wound dressings.

For future perspectives, the spreadability, as well as the mechanical and rheological behavior of the materials, will be optimized in view of their use as suitable bioinks for the design of 3D printed patches/wound dressings with tailored physico-chemical and mechanical properties for skin regeneration.

## Figures and Tables

**Table 1:** Nomenclature and quali-quantitative chemical composition of the different formulations.

Constituents	Topical Formulations			
	I	II	III	IV
**Bioactive Compounds**				
Mammalian gelatin	0.5 g	-	-	-
Marine collagen from *Rapana venosa*	-	0.5 g	-	-
Marine gelatin from *Rapana venosa*	-	-	0.5 g	-
Collagen hydrolysate from *Rapana venosa*	-	-	-	0.5 g
*Cladophora vagabunda* extract	1.0 g	1.0 g	1.0 g	1.0 g
Grape Pomace extract	1.0 g	1.0 g	1.0 g	1.0 g
**Polymer Matrix**				
			
Carbopol 940	0.5 g	0.5 g	0.5 g	0.5 g
Triethanolamine	0.75 g	0.75 g	0.75 g	0.75 g
Citric acid	0.75 g	0.75 g	0.75 g	0.75 g
Water *quantum satis* up to	100 mL	100 mL	100 mL	100 mL

**Table 2:** Biochemical parameters of marine sources (correlated to the dry substance, DS %).

Source	Dry Substance (DS) (%)	Humidity	Ash-Mineral Substance (%)	Protein Content (%)
***Rapana venosa***	28.87	69.50	2.25	56.87
***Cladophora vagabunda***	7.49	68.10	3.95	38.79

**Table 3:** Results highlighting organic fraction and inorganic residue at 600 °C from Thermogravimetric Analysis (TGA) and antioxidant activity electrochemical (AA%). Standard uncertainty of TGA measurements was 0.5% w, while the SD for the electrochemical measurements was 4.5% (*n* = 3).

Formulation	Organic Fraction (%)	Inorganic Residue (%)	AA (%)
**I**	19.8	1.5	20.0
**II**	17.7	0.8	44.67
**III**	21.5	0.2	52.49
**IV**	17.3	0.2	34.0

**Table 4:** Diameter of microbial growth inhibition zone (mm) by using the four different formulations. Experimental data are reported as mean ± SD (*n* = 3).

Formulation	Diameter of Growth Inhibition Zone (mm)		
	*Staphylococcus aureus*	*Escherichia coli*	*Candida albicans*
**I**	15.9 ± 1.7	17.0 ± 1.4	9.0 ± 2.4
**II**	16.8 ± 1.2	17.5 ± 2.2	9.5 ± 2.3
**III**	17.3 ± 2.8	18.2 ± 1.4	9.5 ± 1.7
**IV**	17.3 ± 2.7	18.4 ± 2.9	9.0 ± 1.7

## Supplementary Materials

The following supporting information can be downloaded at: https://www.mdpi.com/article/10.3390/ma15103507/s1, Figure S1. Results from (**A**) Thermogravimetric Analysis (TGA), (**B**) Differential Thermal Analyses (DTA) and (**C**) the first derivative of the TGA signal (dTG) of the Carbopol 940, formulation I and III, dried at 40 °C for 14 days. Figure S2. Cyclic Voltammetry (CV) of Carbopol 940.
