# Supplementary material for: Marine and Agro-Industrial By-Products Valorization Intended for Topical Formulations in Wound Healing Applications
Source: Materials (Basel). 2022 May 13;15(10):3507. doi: 10.3390/ma15103507 (PMC9143632; doi:10.3390/ma15103507)
Supplement: Supplementary file 1 [file materials-15-03507-s001.zip › materials-1702122-supplementary.pdf]

materials

MDPI

Article

Marine and Agro-Industrial By-Products Valorization Intended for Topical Formulations in Wound Healing Applications

Ana-Maria Prelipcean [1], Andreea Iosageanu [1], Alexandra Gaspar-Pintiliescu [1], Lucia Moldovan [1], Oana Craciunescu [1], Ticuta Negreanu-Pirjol [2], Bogdan Negreanu-Pirjol [2], Raul-Augustin Mitran [3], Mariana Marin [3] and Ugo D'Amora [4,*]

[1] National Institute of R&D for Biological Sciences, 296 Splaiul Independentei, Bucharest, 060031, Romania; annastanciuc@gmail.com (A.-M.P.); andreea.iosageanu@gmail.com (A.I.); alex.gaspar@yahoo.com (A.G.-P.); moldovanlc@yahoo.com (L.M.); oana_craciunescu2009@yahoo.com (O.C.)

[2] Faculty of Pharmacy, University of Constanta, University "Ovidius" of Constanta, 1 Aleea Universității, 900470 Constanta, Romania; ticuta_np@yahoo.com (T.N.-P.); bnegreanupirjol@yahoo.com (B.N.-P.)

[3] "Ilie Murgulescu" Institute of Physical Chemistry, Romanian Academy, 202 Splaiul Indepedentei, Bucharest, 060021, Romania; raul.mitran@gmail.com (R.A.-M.); mariana_marin12@yahoo.com (M.M.)

[4] Institute of Polymers, Composites and Biomaterials, National Research Council, Naples, 80125, Italy

* Correspondence: ugo.damora@cnr.it (U. D.)

Abstract: Over the past years, the research attention has been more focusing on waste-derived, naturally-derived and renewable materials, in the view of a more sustainable economy. In this work, different topical formulations were obtained from the valorization of marine and agro-industrial by-products and the use of Carbopol 940 as gelling agent. In particular, the combination of extracts obtained from the marine snail, *Rapanosa venosa,* with *Cladophora vagabunda* and grape pomace extracts, was investigated for wound healing purposes. *Rapana venosa* has demonstrated wound healing properties and antioxidant activity. Similarly, grape pomace extracts have showed to accelerate the healing process. However, their synergic use has not been explored yet. To this aim, four different formulations were produced. Three formulations differed for the presence of a different extract of *Rapana venosa*: marine collagen, marine gelatin and collagen hydrolysate, while another formulation used mammalian gelatin, as further control. Physico-chemical properties of the extracts as well as of the formulations were analyzed. Furthermore, thermal stability was evaluated by thermogravimetric analysis. Antioxidant capacity and biological behavior, in terms of cytocompatibility, wound healing and antimicrobial potential were assessed. The results highlighted for all the formulations *(i)* a good conservation and thermal stability in time, *(ii)* a neutralizing activity against free radicals, *(iii)* and high degree of cytocompatibility and tissue regeneration potential. In particular, collagen, gelatin and collagen hydrolysate obtained from the *Rapana venosa* marine snail represent an important, valuable alternative to mammalian products.

Keywords: *Rapana venosa* compounds; *Cladophora vagabunda*; grape pomace; marine and agro-industrial by-products valorisation; wound healing; topical formulations

Citation: Prelipcean, A.-M.; Iosageanu, A.; Gaspar-Pintiliescu, A.; Moldovan, L.; Craciunescu, O.; Negreanu-Pirjol, T.; Negreanu-Pirjol, B.; Mitran, R.-A.; Marin, M.; D'Amora, U. Marine and Agro-Industrial By-Products Valorisation Intended for Topical Formulations in Wound Healing Applications. *Materials* **2022**, *15*, 3507. https://doi.org/10.3390/ma15103507

Academic Editor: Wenxin Wang

Received: 12 April 2022
Accepted: 11 May 2022
Published: 13 May 2022

Publisher's Note: MDPI stays neutral with regard to jurisdictional claims in published maps and institutional affiliations.

Copyright: © 2022 by the authors. Licensee MDPI, Basel, Switzerland. This article is an open access article distributed under the terms and conditions of the Creative Commons Attribution (CC BY) license (https://creativecommons.org/licenses/by/4.0/).

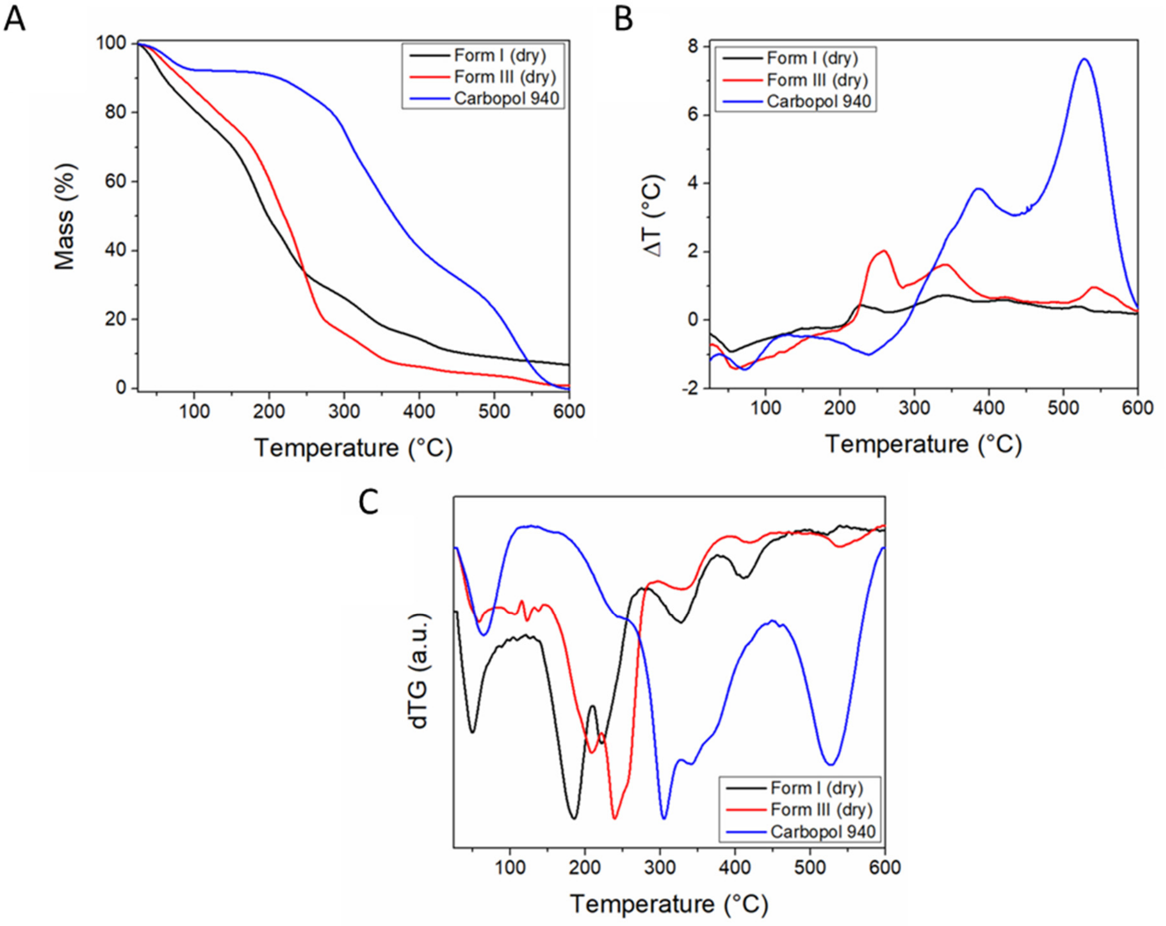

Figure S1. Results from (**A**) Thermogravimetric Analysis (TGA), (**B**) Differential Thermal Analyses (DTA) and (**C**) the first derivative of the TGA signal (dTG) of the Carbopol 940, formulation I and III, dried at 40 °C for 14 days.

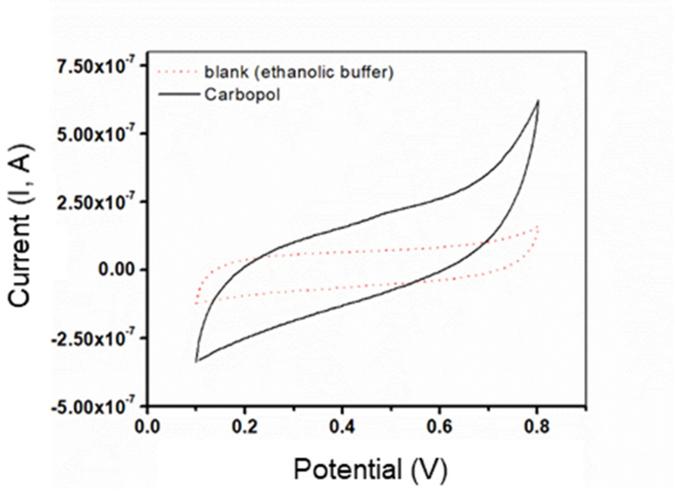

Figure S2. Cyclic Voltammetry (CV) of Carbopol 940.
